# A UK validation of a general measure of subjective well-being: the modified BBC subjective well-being scale (BBC-SWB)

**DOI:** 10.1186/1477-7525-11-150

**Published:** 2013-09-03

**Authors:** Eleanor Pontin, Matthias Schwannauer, Sara Tai, Peter Kinderman

**Affiliations:** 1 Institute of Psychology, Health and Society, University of Liverpool, Waterhouse Building, Liverpool L69 3GL, UK; 2 Clinical Psychology, School of Health in Social Science, Teviot Place, Edinburgh EH8 9AG, UK; 3 School of Psychological Science, 2nd Floor Zochonis Building, Brunswick Street, Manchester M13 9PL, UK

**Keywords:** Subjective well-being, Measurement, Confirmatory factor analysis, Validation

## Abstract

**Background:**

The BBC Subjective Well-being scale (BBC-SWB) is a recently developed questionnaire designed to measure people’s subjective experiences across the wide breadth of domains commonly included in definitions of well-being. Although it has previously been shown to be a reliable and valid measure of subjective well-being in the general population with good psychometric properties, a limitation of the initial version was that it was developed using responses on a 4-point Likert-style scale. This paper presents the psychometric properties, validity and reliability of a revised version of the scale conducted using 5-point Likert-style responses and tests the hypothesis that the scale measures three underlying dimensions of well-being; psychological; physical health; and relationships.

**Methods:**

A sample of 23,341 participants completed the revised BBC-SWB as part of an on-line open-access battery of self-report measures. Confirmatory factor analysis was used to test the pre-hypothesised three factor structure, and internal consistency was investigated using Cronbach’s alpha. Concurrent validity was assessed through analysis of correlations with demographic variables, scores on the Goldberg Anxiety and Depression Scales, and the List of Threatening Experiences Questionnaire.

**Results:**

Confirmatory factor analysis supported three factor structure of the measure in the whole sample and for subsamples of males and females. Both the total 24-item scale and the three subscales had good internal consistency, showed no evidence of floor and ceiling effects and correlated significantly with measures of concurrent validity.

**Conclusions:**

This study provided further confirmation of the validity and utility of the BBC Subjective Well-being scale. The modified version is a reliable and valid measure for the online assessment of subjective well-being in the general population with good psychometric properties.

## Background

It is widely accepted that well-being is a multidimensional concept encompassing multiple domains of human functioning [1]. It is perhaps best defined as a state ‘in which the individual is able to develop in their potential, work productively and creatively, build strong and positive relationships with others, and contribute to their community’ [2]. The term ‘well-being’ encompasses several different concepts, and touches on issues of life satisfaction, social functioning and more practical aspects of quality of life [3-6]. It is perhaps useful to separate the notion of one’s subjective well-being from objective or external factors that drive or influence well-being such as material (e.g. housing) and financial (e.g. income) factors. Subjective well-being concerns peoples’ self-reported assessment of their own well-being;- an individual’s appraisal of a person’s environmental circumstances, a person’s behavioural response and the subjective consequences of that process [7]. Subjective indicators of well-being refer to questions which ask individuals about feelings, experiences and how they evaluate their lives as a whole [7], and are in contrast to the more traditional approach which uses objective indicators such as educational attainment, health, and employment to determine well-being [8]. Objective indicators fail to take account of human perception which is fundamental to understanding an individual’s well-being [9].

Objective and subjective well-being is fundamental to many international economic strategy recommendations and the measurement and monitoring of both is increasingly required for policy development and evaluation [7,10]. Well-validated and reliable measurement of subjective well-being is, therefore, now of central importance in economics and the social sciences [11]. There are numerous existing measures of well-being and related concepts, although they have tended to address particular aspects of well-being rather than incorporating a full spectrum of domains of well-being, or have been developed for very specific purposes and thus have limited application in general population settings. Two examples are the well-established WHOQOL [12], WHOQOL-BREF [13] and the Euroqol [14], which assess well-being with more of a focus on well-being in relation to physical health status. In addition, measures of subjective well-being have been developed such as the Diener scale [15] which focuses on beliefs and attitudes related to well-being, and the Lyubomirsky scale [16] which assesses an individual’s sense of comparison with peers; although they fail to serve as adequate replacements to measures of general well-being [17]. As such, researchers have required new assessment tools to measure well-being. Two examples are the Psychological Well-being Questionnaire [18], which assesses well-being on six subscales; self-acceptance, positive relations with others, autonomy, environmental mastery, purpose in life and personal growth, and the other the Warrick-Edinburgh Mental Well-being Scale (WEMWBS) [19], which focuses solely on positive psychological functioning. Whilst, these two measures offer a more detailed exploration of subjective general well-being and include the psychological and social domains that were under-emphasised in the WHOQOL-BREF and the Euroqol, they suffer from the commensurate weakness of lacking emphasis on those physical aspects of well-being [17].

To prevent the inevitable choice when measuring well-being between the more physical and environmental focus of the WHOQOL-BREF and the more subjective and psychological focus of the WEMWBS, The BBC Subjective Well-being scale (BBC-SWB) (previously named the BBC Well-being Scale) [17] was recently developed to provide a measure of general wellbeing to combine these broad approaches and to incorporate the wide breadth of domains commonly included in the definition of well-being in a format simple enough to be used in a wide range of research and clinical settings [17].

As reported previously [17], items for the 24-item self-report questionnaire were selected from several existing established measures of well-being [13,18] chosen to measure the wide breadth of domains commonly included in the definition of well-being [1,7] and supplemented by additional questions commonly used in mental health settings. Items were chosen to reflect the four domains (physical health, psychological health, social relationships and environment) of the WHOQOL-BREF [13], and the six domains (self-acceptance, autonomy, environmental mastery, purpose in life, positive relations with others and personal growth) of the Psychological Well-Being Questionnaire [18]. In addition, supplementary questions were generated by the authors to reflect the ‘negative cognitive triad’ of thoughts about self, world and future derived from the dominant psychological model of low mood [20].’

The BBC-SWB has already been shown to be a reliable and valid measure of subjective well-being in a general population sample with good psychometric properties [17]. However, a limitation of the initial version was that it was developed using responses on a 4-point Likert-style scale. Four-point Likert scales have been found to be unfavourable both in terms of the extent that they allow participants adequately to express their response to questionnaire items [21], and in how responses are treated as interval data required for certain statistical analysis. In contrast, 5-point Likert scales are more closely approximated to interval data and have been found to improve data quality, internal consistency and discriminant validity [22].

The present study aims to validate the modified 5-point Likert style response version of the BBC-SWB in an adult UK population, and to determine if the psychometric properties demonstrated in the previous validation study are replicated in sub-group samples of males and females.

## Methods

### Measure

The revised BBC Well-being (The BBC-SWB) was included in an open-access battery of self-report measures named ‘The Stress Test’. The Stress Test was a major on-line investigation of the social, environmental and psychological determinants of mental ill-health conducted in collaboration with BBC Lab UK [23]. The results of the wider study will be reported elsewhere. The BBC-SWB comprised 24 items hypothesised to reflect three underlying dimensions; ‘psychological well-being’; ‘physical health and well-being’; and ‘relationships’. These comprised the three subscales of the measure (Table 1). Participants completing the scale were instructed that the questionnaire ‘attempts to measure how happy you feel generally in most parts of your life’. In contrast to the previous version of the scale, where participants were required to select one of four options, in the revised version, respondents were required to select their answer from one of five options that best describes their experience. These were; ‘not at all’ (1); ‘a little’ (2); ‘moderately’ (3); ‘very much’ (4); and ‘extremely’ (5). All items except one were scored positively from one to five, with five reflecting greater well-being. One item, asking about anxiety and depression, was reversed scored.

**Table 1:** Descriptive statistics for the BBC subjective well-being scale, n = 23,341

	***M***	***SD***
V1. Are you happy with your physical health	2.96	1.014
V2. Are you happy with the quality of your sleep	2.71	1.103
V3. Are you happy with your ability to perform daily living activities	3.29	1.020
V4. Do you feel depressed or anxious	3.46	1.073
V5. Do you feel able to enjoy life	3.17	0.968
V6. Do you feel you have a purpose in life	3.09	1.171
V7. Do you feel optimistic about the future	3.03	1.105
V8. Do you feel in control of your life	2.89	1.062
V9. Do you feel happy with yourself as a person	2.97	1.047
V10. Are you happy with your looks and appearance	2.72	0.976
V11. Do you feel able to live your life the way you want	2.74	1.065
V12. Are you confident in your own opinions and beliefs	3.61	0.980
V13. Do you feel able to do the things you choose to do	3.08	0.974
V14. Do you feel able to grow and develop as a person	3.14	1.065
V15. Are you happy with yourself and your achievements	3.11	1.024
V16. Are you happy with your personal and family life	3.27	1.094
V17. Are you happy with your friendships and personal relationships	3.21	1.031
V18. Are you comfortable about way you relate and connect with others	3.12	1.016
V19. Are you happy with your sex life	2.49	1.258
V20. Are you able to ask someone for help with a problem	2.92	1.150
V21. Are you happy that you have enough money to meet your needs	3.03	1.174
V22.Are you happy with your opportunity for exercise/leisure	3.02	1.137
V23. Are you happy with access to health services	3.54	0.948
V24. Are you happy with your ability to work	3.48	1.045

*Note*. Variable 4 was reverse scored. All items rated on a 5-point scale, 1 = never to 5 = almost always.

The study was approved by the University of Liverpool, UK, Research Ethics Committee and was conducted in accordance with ethical standards of the 1964 Declaration of Helsinki. All respondents were required to give informed consent prior to their participation and were required to sign in using a BBC online membership username and password. Answers were selected from a drop-down menu and once the test completed, participants were not permitted to compete the test again. Respondents from 165 countries participated in The Stress Test (*N* = 32,827). Of these, 82.7% (*n* = 27,138) of eligible respondents (age 18–85 years) were from the UK. The present analysis was conducted on UK citizens only because it is well known that factors such as income, healthcare, social services and even political governance all affect quality of life and subjective well-being [11]. While comparison of subjective well-being between residents of different countries is an entirely valid enterprise, it is more appropriate to establish the psychometric validity of measures within defined populations.

### Validation measures

For the purpose of scale validation, and to replicate the previous validation study [17], data from the BBC-SWB were analysed alongside demographic variables and two additional measures from The Stress Test battery. Demographic variables were; age, gender, highest level of formal schooling, gross annual household earnings, and occupational status. The two additional measures were; the Goldberg Anxiety and Depression Scales (GADs) [24] and the List of Threatening Experiences Questionnaire (LTE-Q) [25]. The GADs are an 18-item self-report symptom inventory with yes/no responses to anxiety and depression symptomatology. Nine items each comprise the anxiety and depression scales and they are considered to be a valid and acceptable method of detecting depression and anxiety in the general population [26]. The LTE-Q records the incidence (composite score) of negative life events including illness or injury, death of a close friend or relative and unemployment in the previous 12 months. It has shown good test-retest reliability and high agreement between participants and informant ratings [25].

### Participants

For the purpose of this study, a sample of 23,341 UK residents with complete data was drawn from the larger dataset of 32,827. The mean age of the sample was 41.8 years (*SD* 13.8 *Min* 18, *Max* 85) and 9112 (39%) were male, 14,229 (61%) female. 22,311 (95.6%) described themselves as ‘White British’, with 25 describing themselves as ‘Asian British’, 151 as ‘Black British’, 176 as ‘Chinese or Chinese British’, 7 as ‘Middle Eastern’, 150 as ‘Mixed White Asian’, 112 as ‘Mixed White Black’, 6 as ‘Mixed other’, 135 as ‘Other ethnic group and 268 indicated they would rather not say.

In this sample, 4911 (21%) described themselves as single, 2097 (9%) as in a relationship but not cohabiting, 13,969 (59.9%) as either married or cohabiting, 377 (1.6%) widowed, and 1987 (8.5%) as divorced or separated.

With regard to the highest level of formal schooling achieved, the majority of the sample (*n* = 10,868, 46.6%) reported being educated to degree level or having a professional qualification, and a further 6240 (26.7%) had a postgraduate degree. In contrast, 471 (2%) participants did complete schooling up to age 16, but 1920 (8.2%) stayed in education until 16, and 3842 (16.5%) completed post-16 education or vocational training to age 18. Three hundred and seventy seven (1.6%) of the sample reported they were still at school and 1668 (7.1%) were at university. 17,737 (71.6%) participants were in full or part time employment or were self-employed. In contrast, 1256 (5.4) were unemployed, 1873 (8%) retired and 430 (1.8%) were doing voluntary work. Participants reported their total gross annual household income was less than £9999 per annum in 1956 (8.4) cases. 3297 (14.1%) reported income of £10,000-£19,999 per annum, 3874 (16.6%) income of £20,000-£29,000 per annum, 3392 (14.5%) income of £30,000-£39,999 per annum, 2625 (11.2%) income of £40,000-£49,999 per annum, 3379 (14.5%) income of £50,000-£74,999, 2766 (11.9%) income of £79,000 or more per annum, while 770 did not know their household income, and 1282 preferred not to say.

### Data analysis

Confirmatory factor analysis (CFA), as implemented in EQS V 6.2 [27], was used to test the pre-hypothesised three factor structure of the BBC-SWB. The CFA was carried out on the whole sample as well as gender sub-samples in order to test measurement invariance across different sub-groups. In a first step we tested the difference of the variance–covariance matrices by comparing relating items in the measure across the groups. In a second step we tested the configural invariance and scalar invariance, by demonstrating that the factors and pattern of factor loadings are not significantly different across the two groups. For this second step we tested a multi group model and its fit parameters.

Prior to analysis, data were checked for univariate and multivariate normality. Univariate normality was determined for each variable through examination of skewness and kurtosis. Multivariate normality was assessed using Mardia’s Coefficient [28], which evaluates multivariate normality through evaluation of multivariate kurtosis. In accordance with most social and behavioural science data [29], examination of the Mardia’s coefficient suggested non-normality in the population therefore for the CFA model robust maximum-likelihood estimation (which adjusts the standard errors and provides the Satorra-Bentler chi-square) was employed.

Goodness of fit of CFA models was evaluated using the Satorra-Bentler robust fit statistics; The Satorra-Bentler χ^2^ (S-B χ^2^) and the Robust Comparative Fit Index (RCFI) [30]. The chi-squared is the most commonly used measure of model fit and assesses the model’s ‘badness of fit’ such that a high chi-squared value with a significant *p* value is suggestive of a poor fit of the model to the data. However, because the sample size in this study was very large, a significant chi-squared was expected. In addition to the model χ^2^, the RCFI was also used to estimate overall and incremental model fit. The RCFI signifies where the estimated model lies on a continuum of model fit (one end is the independence model where variables are completely uncorrelated, the other a model of perfect correlation). A value of .00 indicates no fit, and 1.0 perfect fit. The criterion of RCFI greater than .90 was used as an indicator of acceptable model fit [31]. We further report the Root Mean Square Error of Approximation (RMSEA) [32], which is a measure of fit per degrees of freedom and compares the lack of fit compared to a perfect model, controlling for sample size. RMSEA values decrease with increasingly good fit with values of 0.06 or less indicative of adequate fit [31]. Estimated correlations between factors were also examined to determine discriminant validity between the three factors. Correlations not exceeding .85 indicate that the factors are measuring different underlying constructs [33].

Internal consistency of the revised scale was investigated using Cronbach’s alpha coefficients [34] and was calculated for the total 24-item scale and each of the three subscales. Total and item scores were examined for floor and ceiling effects. Concurrent validity was assessed through analysis of correlations with the selected demographic variables and the GADs and the LTE-Q scores. CFA analysis was conducted using EQS Version 6.2 [17]. All other analyses were conducted in SPSS Version 20 [35].

## Results

### Construct validity

**Figure 1:**
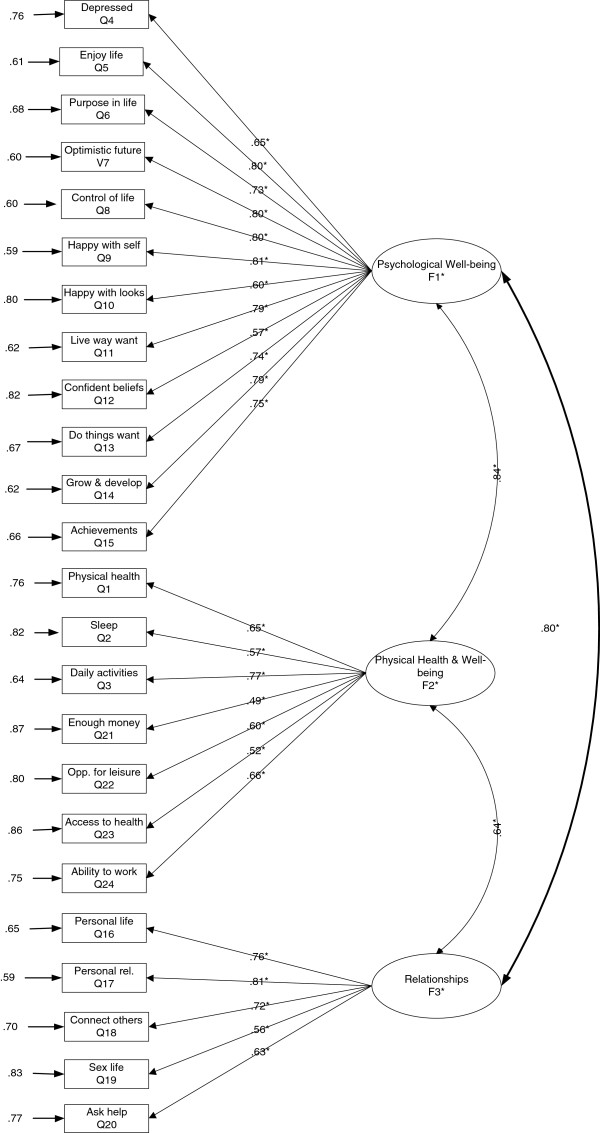
**Confirmatory factor model for the three-factor 24-item BBC-SWB showing completely standardised robust maximum likelihood parameter estimates fitted to the full sample, *n*** = 23,341 All coefficients are statistically significant, *p **< .001.** The figure shows the latent factors (represented by ovals) of ‘psychological well-being’ ‘physical health and well-being’ and ‘relationships’. The questionnaire items (measured variables) are represented by rectangles. The numbers on the straight arrows connecting the latent factor to the questionnaire item are the loadings of items onto the latent factors, and the numbers by the straight arrows going towards the questionnaire items are the error in the measured variable not accounted for by the factor. The double headed arrow between the latent factors depicts the correlations (standardised covariances) between the latent constructs.

In previous research, a three-factor structure for the original BBC well-being scale was shown to be superior to a two-factor and four-factor model [9]. After rejection of a one-factor model in this sample of the revised measure; S-B χ^2^ (252, N = 23, 341) = 39561.6, *P* < 0.001; RCFI = .857; RMSEA = .082 (.081-.082), a three factor CFA model was hypothesised for the structure of the revised version and was performed on the whole sample. It was hypothesised that; factor 1 (Psychological Well-being) predicts items 4 to 15; factor 2 (Physical Health and Well-being) predicts items 1, 2, 3, 21, 22, 23, and 24, and factor 3 (Relationships) predicts items 16 to 20 (see Figure 1). Each indicator (questionnaire item) was constrained to load onto the factor it was designated to measure and residual terms for all indicators were fixed to be uncorrelated. Factor covariances were free to be estimated. For the whole sample, the hypothesised model yielded an acceptable fit of the data; RCFI = .910 and RMSEA = .065 (.065-.066), although the SB-χ^2^ was significant (S-B χ^2^ (249, N = 23, 341) = 25130.9, *P* < 0.001). However, χ^2^ is extremely sensitive to sample size, such that small variations in fit can result in statistically significant and sizable χ^2^. All items were significantly associated with their respective factor and loadings ranged from 0.60 to 0.80 for the ‘psychological well-being’ factor, 0.49 to 0.77 for the ‘physical health and well-being’ factor, and 0.56 to 0.81 for the ‘relationships’ factor. The three factor model including significant coefficients in standardised form is shown in Figure 1.

Correlations between factors revealed strong associations between factors; F1 (Psychological Well-being) and F2 (Physical Health and Well-being) were significantly correlated, *r* = .839, *p* < .001; F1 and F3 (Relationships), *r* = .804, *p* < .001; and F2 and F3, *r* = .640, *P* < .001, which indicate them to be distinct but highly correlated constructs. Given the high correlation between F1 and F2, these factors were collapsed into a single factor in order to test a competing two-factor model. However this revealed a worse fit of the data, (S-B χ^2^ = (251, N = 23, 341) = 39561.6, *P* < 0.001; RCFI = .857; RMSEA = .082 (.081-.082). In addition, a second-order model with three first-order factors as indicators was tested. Whilst this revealed an acceptable fit of the data; (S-B χ^2^ = (249, N = 23, 341) = 25204.8, P < 0.001; RCFI = .909; RMSEA = .066 (.065-.066), it was not as good fit as the hypothesised three-factor model.

The three-factor model was tested for invariance to determine measurement and structural equivalence of the BBC-SWB across gender. A multi group model was tested across male (*n* = 9112) and female participants (*n* = 14,229). The multi group model of the two samples demonstrated acceptable fit statistics, SB-χ^2^ = 23557.056, *P* < 0.001, RCFI = .915, RMSEA = .062 (.061-.063), thus demonstrating equivalence of parameters and factor correlation across male and female sub populations.

### Internal consistency

Cronbach’s alpha coefficients calculated using the whole sample (*n* = 23,341) revealed high levels of internal consistency for the total 24-item scale (Cronbach’s alpha = .944, 24 items), the ‘psychological well-being’ scale (Cronbach’s alpha = .934, 12 items), the ‘physical health and well-being’ scale (Cronbach’s alpha = .801, 7 items), and for the ‘relationships’ scale (Cronbach’s alpha = .816, 5 items). These were replicated across subsets of the population (Table 2) although a Cronbach’s alpha coefficients were very slightly lower for those over 75 years, and in the depressed and anxious populations for the total 24-item scale and for the three subscales.

**Table 2:** Descriptive statistics and Cronbach’s alpha coefficients across subscales in subsets of the population

		**Total well-being score**		**Psychological well-being subscale**		**Physical health and well-being subscale**		**Relationships subscale**	
	**n**	**M (SD)**	**α**	**M (SD)**	**α**	**M (SD)**	**α**	**M (SD)**	**α**
Gender									
Male	9112	74.2 (17.0)	.94	37.3 (9.6)	.93	22.2 (5.0)	.80	14.7 (4.3)	.82
Female	14,229	73.9 (16.8)	.94	36.8 (9.5)	.93	21.9 (5.0)	.80	15.2 (4.2)	.81
Age									
18–34 years	8020	74.7 (17.0)	.94	37.3 (9.7)	.93	22.0 (5.1)	.80	15.3 (4.3)	.81
35–54 years	10,433	72.8 (16.8)	.95	36.3 (9.5)	.94	21.7 (5.0)	.80	14.8 (4.2)	.82
55–74 years	4767	75.7 (16.4)	.95	37.9 (9.2)	.93	22.7 (4.9)	.81	15.0 (4.1)	.82
75 years and above	121	74.9 (14.9)	.93	38.0 (8.2)	.91	22.1 (4.9)	.81	14.8 (3.6)	.74
Ethnic group									
White	22,311	74.1 (16.8)	.94	37.0 (9.5)	.93	21.5 (5.4)	.80	15.0 (4.2)	.82
Black minority	762	72.8 (17.8)	.94	36.9 (10.1)	.93	22.1 (5.0)	.82	14.5 (4.4)	.82
Ethnic									
Educational level									
‘A Levels’ (Age 18) or equivalent	6233	70.3 (17.8)	.95	35.0 (10.1)	.94	20.8 (5.3)	.81	14.5 (4.4)	.81
Degree/Professional qualification	17,108	75.4 (16.3)	.94	37.7 (9.2)	.93	22.5 (4.9)	.80	15.2 (4.2)	.82
Occupational status^*^									
Employed	17,563	74.5 (16.5)	.94	37.9 (9.4)	.93	22.2 (4.8)	.90	15.1 (4.2)	.82
Unemployed	1252	62.4 (18.0)	.94	30.9 (10.0)	.93	18.2 (5.6)	.81	13.3 (4.4)	.81
Relationship status									
In a relationship	16,066	75.6 (16.4)	.94	37.6 (9.3)	.93	22.2 (5.0)	.80	15.8 (4.0)	.81
Single	7275	70.5 (17.1)	.94	35.6 (9.8)	.93	2.0 (5.2)	.81	13.4 (4.1)	.80
Mental health									
Depressed^**^	5074	58.6 (13.7)	.91	28.3 (7.7)	.89	18.0 (4.5)	.72	15.8 (4.0)	.78
Non-depressed	18,267	78.9 (15.0)	.93	39.4 (8.5)	.92	23.2 (4.6)	.77	16.2 (3.9)	.80
Anxious^**^	8079	63.6 (15.1)	.93	31.5 (8.7)	.92	18.8 (4.6)	.74	13.3 (4.1)	.79
Non-anxious	15,262	79.6 (15.0)	.93	39.9 (8.6)	.91	23.7 (4.4)	.76	15.9 (4.1)	.80

^*^Of those of working age.

^**^Score of > 6 on the Goldberg Anxiety and Depression Scales.

### Distribution

**Figure 2:**
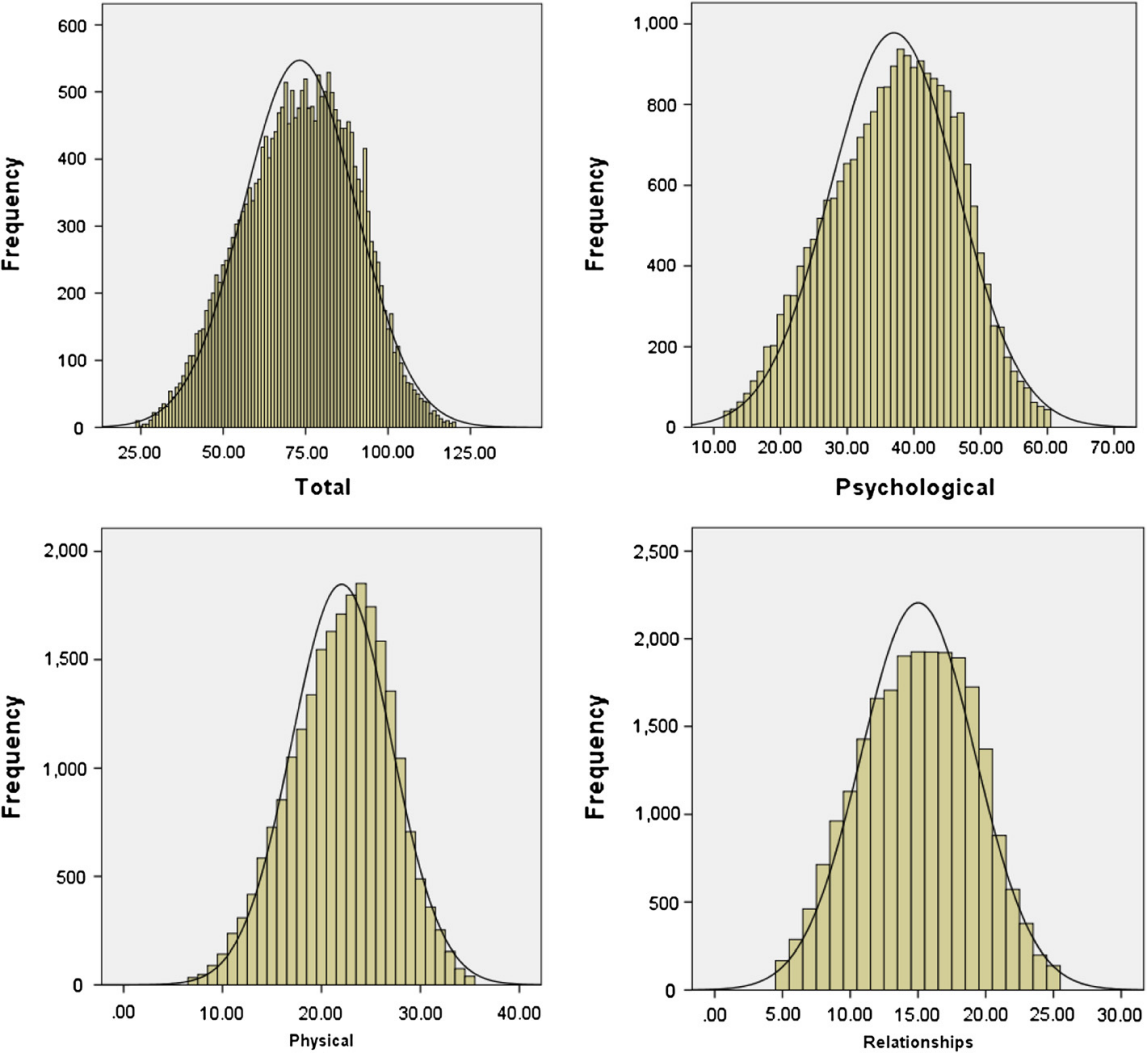
Score distribution for the total BBC-SWB, and the three subscales; ‘psychological well-being’ ‘physical health and well-being’ and ‘relationships’.

Examination of univariate and multivariate normality for each questionnaire item was highly suggestive of non-normal distributions in the population. This is entirely to be expected in measures of this kind. However, the observed distributions of the total scale and all three subscales appeared normally distributed (see Figure 2). Whilst the Kolmogorov–Smirnov Z scores for deviation from normality were statistically significant in each case, this is likely to be an artifact of the very large sample size. Neither the main scores nor any of the subscale scores showed evidence of floor or ceiling effects (see Figure 2).

For the total scale, the mean score for the whole sample was 73.21 (*Median* = 74; *SD*=17.02, *Min* 24, *Max* 120, interquartile range 62-86); mean score for the subscale ‘psychological well-being’ was 36.99 (*Median* = 38; *SD* = 9.52, *Min* 12, *Max* 60, interquartile range 30-44), mean score for ‘physical health and well-being’ was 22.03 (*Median* = 22; *SD* = 5.04, *Min* 7, *Max* 35, interquartile range 19-26; and mean score for ‘relationships’ subscale was 15.01 (*Media*n = 15; *SD* = 4.22, *Min* 5, *Max* 25, interquartile range 12-18). It is notable that the median and mean scores were, in each case, extremely similar.

### Concurrent validity

Correlations with the Goldberg Anxiety and Depression Scales, and the composite score of the List of Threatening Experiences, revealed that anxiety, depression, and the number of negative life events are negatively correlated with the total well-being scale and the three subscales (Table 3).

**Table 3:** **Correlations between the BBC-SWB and measures from The Stress Test, *n*** **= 23,341**

**Subscale variable**	**Total**	**Psychological well-being**	**Physical health and well-being**	**Relationships**
Goldberg anxiety scale	*r* = −.588	*r* = −.542	*r* = −.576	*r* = −.375
	*P* < .0005	*P* < .0005	*P* < .0005	*P* < .0005
Goldberg depression scale	*r* = −.661	*r* = −.642	*r* = −.589	*r* = −.456
	*P* < .0005	*P* < .0005	*P* < .0005	*P* < .0005
Number of negative life events	*r* = −.237	*r* = −.208	*r* = −.271	*r* = −.159
	*P* < .0005	*P* < .0005	*P* < .0005	*P* < .0005

## Discussion

This study provided further confirmation of the validity and potential utility of the modified BBC-SWB. Confirming initial results obtained in the development and validation of the first version [17], analysis of the current data of UK citizens in a very large on-line general population sample showed that the modified scale performs extremely well as a general measure of well-being. The scale has good face validity, very good internal consistency across subsets of the sample, and good concurrent validity. Confirmatory factor analysis supported the hypothesised three-factor structure of the measure following the rejection of a single factor and two-factor model. Analysis revealed high correlations between the factors. Although a second-order model with three first-order factors as indicators also revealed an acceptable fit of the data, the three-factor model remained the best fit of the data thus demonstrating good discriminant validity between the subscales relating to underlying dimensions of ’psychological well-being’, ‘physical health and well-being’ and ‘relationships’. The implication as such, is that the three domains of well-being are distinct but highly correlated constructs. This is not necessarily a weakness of the measure, as it reflects the interrelated nature of well-being which encompasses multiple domains.

The measure also demonstrated equivalence of fit and parameters across both the male and female subsamples, therefore demonstrating validity of use of the measure across both males and females, and in mixed gender groups. Scores on the BBC-SWB and its subscales were well-distributed. For the 24-item total scale and each subscale, the distributions were near-normal, with minimal floor and ceiling effects. An absence of floor and ceiling effects are important in a general measure designed for wide utility across different populations. It is also important to note that the mean was very similar to the median, both for the total scale and subscales. This can have important utility in research and clinical settings. Both the total well-being score, and scores for each subscale demonstrated high internal consistency which was replicated across subsets of the population. The very slightly lower Cronbach’s alpha coefficients for those aged over 75 years and in the depressed and anxious subset may indicate that further validation of the measure is needed in the older population and in clinical groups. Concurrent validity was demonstrated by the high negative correlations with the Goldberg scales of anxiety and depression and the number of negative life events determined by the score on the List of Threatening Experiences Questionnaire.

The present validation study is limited in that the test-retest reliability of the BBC Well-being Subjective scale was not examined. In addition, whilst the measure has shown good psychometric properties in this very large UK general population sample, an online, anonymous, convenience sample is unlikely to very representative of the general population of the UK. Indeed, the demographic characteristics of the participants indicated that they were more likely to be White-British, to have slightly higher earnings, and to be better educated than the general UK population, although they were comparable on other demographic features. It is reasonable to conclude therefore that the BBC Subjective Well-being scale is well validated for the UK general population, but further research into its applicability in all communities is still required. Future research would validate the measure on different populations and, given its potential clinical utility for assessing subjective well-being in physical and mental health settings, on clinical samples.

It is acknowledged that, as worded, the scale may be subject to pressures of social desirability, with a clear ‘correct’ or ‘happy’ response. In addition, all the well-being scales were strongly correlated to depression and anxiety, especially the Psychological well-being scale. This may indicate that well-being is strongly driven by psychological health. An individual’s mood may have a significant impact on their evaluations of their well-being which may cause subjective well-being to differ greatly from their objective well-being. However, this can be considered strength of the measure. Rather than only assessing objectively an individual’s physical and social functioning from a set of external circumstances, which assume that certain things improve or detract from an individual’s well-being [10], this measure captures one’s own subjective appraisal of these areas. One’s own perception is fundamental to understanding genuine well-being, both at an individual level and at a National level alongside objective indicators such as material wealth, life expectancy, and child mortality rates [11].

## Conclusion

To conclude, the findings from this study indicate that the modified version of the BBC Subjective Well-being scale is a reliable and valid measure for the online assessment of subjective well-being in the general population with good psychometric properties. It is potentially particularly valuable that BBC-SWB has been validated as an online measure. This comprehensive measure of well-being means it has considerable utility as the demand for measures of well-being increases internationally.

## Abbreviations

BBC-SWB: BBC subjective well-being scale; WHOQOL: World Health Organisation quality of life; WHOQOL-BREF: World Health Organisation quality of life brief; Euroqol: European quality of life; WEMWBS: Warrick-Edinburgh mental well-being scale; GADs: Goldberg anxiety and depression scales; LTE-Q: List of threatening experiences questionnaire; CFA: Confirmatory factor analysis; S-B χ2: Satorra-Bentler chi-squared; RCFI: Robust comparative fit index; RMSEA: Root mean square error of approximation.

## Competing interests

The authors declare they have no competing interests.

## Authors’ contributions

The BBC (British Broadcasting Corporation) hosted ‘The Stress Test’ and provided both technical support in its development and publicity. EP participated in the design and coordination of the study, carried out statistical analysis and drafted the manuscript. MS participated in the design of the study, advised on statistical analysis, and helped draft the manuscript. ST participated in the design of the study, and helped draft the manuscript. PK conceived of the study, designed and coordinated the study, and helped draft the manuscript. All authors read and approved the final manuscript.
